# Elevated serum CEA levels are associated with the explosive progression of lung adenocarcinoma harboring EGFR mutations

**DOI:** 10.1186/s12885-017-3474-3

**Published:** 2017-07-14

**Authors:** Yuan Gao, PingPing Song, Hui Li, Hui Jia, BaiJiang Zhang

**Affiliations:** 1 Department of Thoracic Surgery, Shandong Provincial Hospital affiliated to Shandong University, Jinan, Shandong Province 250117 China; 2 Department of Thoracic Surgery, Shandong Tumor Hospital and Institute, Jinan, Shandong Province 250117 China; 3 Department of Medical Oncology, Shandong Tumor Hospital and Institute, Jinan, Shandong Province 250117 China; 4 Department of Thoracic Surgery, Shandong Cancer Hospital and Institute, Jiyan Rd. 440, Jinan, Shandong 250117 People’s Republic of China

**Keywords:** Biomarker, EGFR, EGFR-TKIs, Mutation, Response

## Abstract

**Background:**

Serum carcinoembryonic antigen (CEA) levels are a predictor of epidermal growth factor receptor tyrosine kinase inhibitor (EGFR-TKI) efficacy and are associated with epidermal growth factor receptor (EGFR) gene mutations. However, the clinical significance of plasma CEA level changes during different cycles of target therapy is unknown for lung adenocarcinoma patients with sensitizing EGFR mutations.

**Methods:**

In total, 155 patients with lung adenocarcinoma were enrolled in this retrospective study between 2011 and 2015. EGFR mutations were detected by RT-PCR (real-time quantitative PCR). Plasma CEA levels were measured prior to different EGFR-TKI treatment cycles. Computed tomography (CT) scans were conducted every 2 months to assess the therapeutic efficacy.

**Results:**

Serum CEA concentrations were significantly associated with EGFR mutations (*p* < 0.05). Furthermore, in all patients treated with EGFR-TKIs, the serum CEA levels increased with disease progression (*p* < 0.005). A COX multivariate analysis revealed that CEA levels 16.2 times above normal were associated with early disease progression (HR, 5.77; 95% CI:2.36 ~ 14.11; *p* < 0.001). Based on this finding, a threshold was set at the median time of 8.3 months. Patients with EGFR mutations exhibited a median progression-free survival time of 12.8 months. Serum CEA levels were markedly increased compared to levels measured 4.5 months prior to the changes detected via CT scans for patients resistant to EGFR-TKIs.

**Conclusions:**

Elevated CEA levels during targeted therapy may be a more sensitive predictor of explosive lung adenocarcinoma progression in patients harboring mutant EGFRs compared to traditional imaging methods.

## Background

Lung cancer is the leading cause of cancer-related mortality worldwide, and non-small cell lung cancer (NSCLC) is the most common form of lung cancer. Many NSCLC patients present with an advanced disease stage upon initial diagnosis [1]. Patients with tumors that harbor activating mutations in the epidermal growth factor receptor (EGFR) benefit greatly from treatment with EGFR tyrosine kinase inhibitors (TKIs) compared with patients whose tumors lack these mutations [2–7]. One such EGFR-TKI is the orally administered, targeted agent erlotinib, which inhibits the tyrosine kinase domain of the EGFR. Erlotinib is approved for second-line use based on the positive results of a phase 3 BR.21 trial [8] in which erlotinib improved overall survival (OS) compared with the best supportive care. Erlotinib also has clinical benefits as a first-line therapy for advanced NSCLC. The tumor response rate was 10-20%, and the median survival duration was 10.9-12.9 months in phase 2 studies [9, 10]. However, almost all patients suffered from tumor progression and inevitably became resistant to EGFR-TKIs within 8-12 months (a phenomenon referred to as acquired resistance).

Currently, the standard method of measuring the efficacy of a lung cancer treatment is anatomical imaging, including computed tomography (CT) scanning, which measures the size of malignant lesions before and after treatment. However, the use of anatomical imaging methods to assess treatment efficacy presents a number of drawbacks, the most critical of which is a delay in treatment due to changes in tumor size. Furthermore, the efficacy of targeted drugs is often not reflected by a change in tumor size but rather by changes in cell metabolism. Therefore, the identification of clinical biomarkers in patients with EGFR mutations may prove useful when anatomical analyses are not feasible.

Several serum markers are considered to be prognostic and predictive markers of NSCLC. Among these markers, carcinoembryonic antigen (CEA) is a sensitive and useful tumor marker for cancer diagnosis and prognosis and the assessment of therapy [6–8]. According to recent reports, CEA is a significant predictor of sensitivity and survival in patients treated with gefitinib [9–11]. The present study (1) compared the significance of CEA levels with other clinical characteristics (i.e., age, sex, smoking history, performance status [PS], and CYFRA1-1) and (2) determined whether the serum CEA levels correlated with EGFR-TKI resistance. This correlation would permit the use of CEA as a biomarker in NSCLC patients and would aid in identifying treatment candidates with reversible and irreversible EGFR-TKI resistance and candidates for whom an early intervention with combined chemotherapy and radiotherapy is more appropriate.

## Methods

### Patients

In total, 155 primary lung adenocarcinoma patients, who had been hospitalized at the Department of Respiratory, Oncology and Thoracic Surgery in Shandong Provincial Tumor Hospital between August 8, 2011, and March 8, 2015, were enrolled in this retrospective study. The enrolled patients tested positive for an EGFR mutation and had received EGFR-TKI as a 1st, 2nd, or 3rd line of treatment [6, 12]. Patients with locally advanced (stage IIIB), metastasized (stage IV), or post-surgically relapsed NSCLC were confirmed for EGFR mutations and received either gefitinib 250 mg/d or erlotinib 150 mg/d orally. The clinical stage was determined by the tumor, node, and metastasis (TNM) classification system (7th edition). The following inclusion criteria were utilized for this study: patients had pathologically identified adenocarcinoma; had received an initial therapy (including chemotherapy, surgery or chemoradiotherapy); and had survived for more than 1 month. Histological subclassification was performed according to the World Health Organization classification. A CT scan was performed to assess the tumor size within 28 days prior to initiating treatment and was repeated every 2 months. Serum tumor markers and CT scans were measured simultaneously. All responses were defined according to the Response Evaluation Criteria in Solid Tumors (RECIST) criteria. A response was confirmed at 4 weeks (for a complete or partial response) or 6 weeks (for stable disease) after the first documentation. Other inclusion criteria included an Eastern Cooperation Oncology Group (ECOG) performance status of 0-3 [13]. Patients who had received prior treatment with oral EGFR-TKIs or were allergic and/or intolerant to these drugs were excluded from the study. The progression-free survival (PFS) and overall survival (OS) of the metabolic responders and non-responders were the end points of the study. The baseline patient characteristics are presented in Table 1. This study complied with the guidelines of the local ethics committee.

**Table 1:** Patient characteristics

Characteristic	No. of Patients	Percentage
Sex		
Male	63	34.2%
Female	92	65.8%
Clinical Stage		
I,II	57	36.8%
III,IV	98	63.2%
Smoking history		
Ever	63	34.2%
Never	92	65.8%
Age,y		
< 59	74	47.7%
≧59	81	52.3%
ECOG PS		
0-1	152	98.1%
2-3	3	1.9%
Median age of patients 59y (range,38-81y)		

Patient characteristics

### Measurement of serum tumor marker levels

Serum CEA (normal range: 0-3.4 ng/ml) and CYFRA 21-1 (normal range: 0-3.3 ng/ml) were measured via an electrochemiluminescence immunoassay on an automatic analyzer (Elecsys200; Roche Diagnostics Mannheim, Basel, Switzerland) before TKI treatment.

### Determination of EGFR mutation

EGFR mutation analysis was performed via a fragment analysis using polymerase chain reaction (PCR) and the Cycleave real-time quantitative PCR techniques (SRL Inc., Tokyo, Japan).

### Statistical methods

There were no missing data in our study. We used SPSS 17.0 statistical software (SPSS Inc., Chicago, IL, USA) for data processing. The χ^2^ test, Fisher’s exact test and multivariate logistic regression analysis were used to analyze the associations between EGFR mutations and clinical factors. Survival was estimated using the Kaplan-Meier method. Overall survival was measured as the date of the first course of initial therapy to the date of death or the last follow-up examination. A log-rank test was performed to evaluate significant differences in the overall survival among the groups. *P* values <0.05 were considered significant. A multivariate analysis using the Cox proportional hazards model was used to establish the association between the clinical variables and survival.

## Results

### Patient characteristics

The clinicopathological characteristics of the 155 patients are summarized in Table 1. Ninety-two patients (65.8%) were women, and 92 patients (65.8%) were non-smokers. The patient age ranged from 38 to 81 years (median: 60 years). Fifty-seven patients were classified as pathological stage classes I and II, and 98 patients were classified as III and IV. One hundred fifty-two patients (98.1%) had a PS of 0-1, and three (1.9%) patients had a PS of 2-3. The median OS and PFS were 28.5 and 12.8 months, respectively.

### Relationship between CEA levels and EGFR gene mutations

A single factor χ^2^ test showed that EGFR mutation was associated with gender, age, smoking history, and the serological levels of CEA and CYFRA 21-1 (*p* < 0.05; Table 2). The multivariate logistic analysis revealed that patient gender and serological CEA levels were correlated with EGFR mutation (*p* < 0.05) (Table 3).

**Table 2:** Analysis of EFGR mutation

Clinical Characteristic	Sample(n)	EGFR mutation(n)	χ2	P
Gender				
Female	92	47	3.957	0.047
Male	63	22		
Age				
< 59	74	30	4.230	0.040
≧59	81	39		
Stage				
I,II	57	21	2.150	0.143
III,IV	98	48		
PS Score				
0-1	152	68	0.155	0.694
2-3	3	1		
Smoker				
Ever	63	22	3.957	0.047
Never	92	47		
CEA				
< 3.4 ng/ml	63	21	5.374	0.020
≧3.4 ng/ml	92	48		
Cyfra21-1				
< 3.3 ng/ml	64	44	25.920	0.001
≧3.3 ng/ml	91	25		

A single factor χ^2^ test showed that EGFR mutation was associated with gender, age, smoking history, and the serological levels of CEA and CYFRA 21-1

**Table 3:** Futher analysis of the association of EGFR mutation

Factor		EGFR Mutation	
	OR	P	95%CI
Gender	0.479	0.034	0.243 ~ 0.946
CEA	2.529	0.001	1.283 ~ 4.984

The multivariate logistic analysis revealed that patient gender and serological CEA levels were correlated with EGFR mutation

**Fig. 1:**
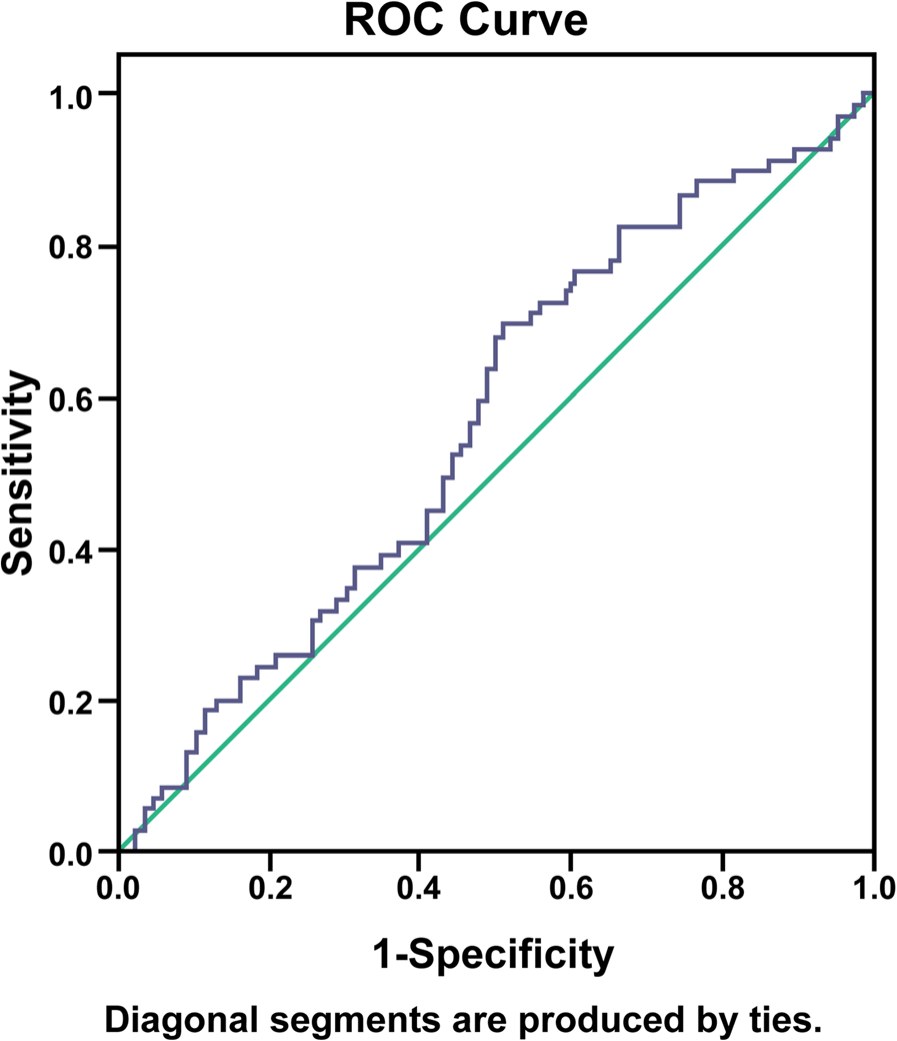
A ROC curve was drawn, and the area under the curve was calculated. The area under the curve for CEA was 0.567 (95% CI: 0.476 ~ 0.657, *p* < 0.001). When the CEA cut-off was 3.4 ng/ml, the sensitivity was 69.6% and the specificity was 48.8%

A ROC curve was drawn, and the area under the curve was calculated. The area under the curve for CEA was 0.567 (95% CI: 0.476 ~ 0.657, *p* < 0.001). When the CEA cut-off was 3.4 ng/ml, the sensitivity was 69.6% and the specificity was 48.8% (Figure 1).

### Association of serum markers and the response to EGFR-TKIs

We evaluated the efficacy of EGFR-TKIs via CT scanning and serum tumor marker concentrations (CEA) every 2 months. The patients were divided according to serum CEA levels into elevated and non-elevated groups in progress group by CT scanning. A χ^2^ test revealed that increased serum CEA levels were related to disease progression (*p* < 0.005). Patients were then divided into two groups according to the average value (CEA levels 16.2 times higher than normal) in elevated group. According to the Cox regression analysis, CEA levels 16.2 times higher than normal at any point during the 2 months prior to the determination of progress represented an early response to disease progression (HR, 5.77; 95% CI:2.36 ~ 14.11; *p* < 0.001; Table 4). We noted that the median time until this threshold (i.e., CEA levels at least 16.2 times higher than normal) was reached was 8.3 months. However, for patients with EGFR mutations, the median was 12.8 months.

**Table 4:** Correlation between CEA levels and disease progression

		B	SE	Wald	df	Sig.	Exp(B)	95.0% CI for Exp(B)	
								Lower	Upper
Step 1	CEA	2.007	.434	21.377	1	.000	7.443	3.178	17.428
Step 2	Age	.701	.355	3.908	1	.048	2.017	1.006	4.043
	CEA	2.041	.444	21.147	1	.000	7.695	3.225	18.361
Step 3	Stage	.855	.428	3.997	1	.046	2.351	1.017	5.435
	Age	.814	.361	5.084	1	.024	2.257	1.112	4.580
	CEA	1.752	.456	14.754	1	.000	5.769	2.359	14.108

According to the Cox regression analysis, CEA levels 16.2 times higher than normal at any point during the 2 months prior to the determination of progress represented an early response to disease progression

## Discussion

EGFR mutation predicts the efficacy of EGFR-TKIs in patients with advanced NSCLC. However, acquiring an adequate tissue sample for an EGFR mutational analysis is not often feasible, particularly in patients with advanced disease [2, 8, 14]. A recent study reported that molecular analyses of circulating tumor cells obtained from the peripheral blood of patients with lung cancer was useful for monitoring changes in epithelial tumor genotype during the course of treatment. However, this type of molecular analysis can be difficult due to the requirement of a specific, microfluidic-based device - the CTC chip. Moreover, there are approximately 486 types of EGFR-TKI domain mutations across 87 species, and new mutations are continually being identified [15, 16]. Recently, the attention moved to the possibility of isolation and analysis of cell-free tumor DNA (cftDNA) that, to date, represents the best candidate for identification and monitoring of molecular tumor-related alterations in blood of patients with cancer [17]. Circulating DNA fragments carrying tumor specific sequence alterations cftDNA are found in the cell-free fraction of blood, representing a variable and generally small fraction of the total circulating DNA. cftDNA has a high degree of specificity to detect *EGFR* gene mutations in NSCLC. Fragments of circulating DNA were isolated in plasma many years ago [18]. In particular, patients with cancers present higher levels of circulating DNA comparing to healthy volunteers because of the presence of tumoral counterpart, which express the same molecular abnormalities expressed by DNA of primitive mass [19]. The elevate cellular turn over and consequent cellular necrosis and apoptosis cause a massive release of tumoral DNA into the bloodstream were it can be isolated and analyzed. Therefore, tumor size, localization and vascularity may influence cftDNA plasmatic levels. It is also possible that part of cftDNA comes from CTCs lysis [19]. The analysis of cftDNA, defined as liquid biopsy, could be repeated every time needed and without any discomfort for patients. Moreover, the mutational analysis of cftDNA demonstrated a signicantly better sensitivity if compared with CTCs one, establishing cftDNA as the best circulating source for molecular analysis [20]. Information derived from liquid biopsy could be used in future for early cancer diagnosis, assessment of genetic determinants for targeted therapies, monitoring of tumor dynamics and early evaluation of tumor response, identification of resistance mechanisms [19]. cftDNA could be a relevant biomarker to molecular diagnosis and monitor treatment resistance, because of its sensitivity and specificity, but it really needs reproducible and standardized methods, both for the extraction and for its analyses. Regarding the mutation analysis of cftDNA, a large number of technologies is now available to analyze mutations in cftDNA, including automatic sequencing, real-time polymerase chain reaction (PCR) platforms, mass spectrometry (MS) genotyping, ampli cation protocols with magnetic beads in oil emulsions [beads, emulsion, ampli cation and magnetics (BEAMing)] and next-generation sequencing (NGS), digital PCR platforms [21–25]. The sensitivity range of the available techniques varies from 15 to 0.01%, but one of the major gaps in this field is the lack of standardization of techniques, in order to understand how those techniques are cost-effective and reliable to the clinical needs.

Therefore, simpler and more accessible predictors of EGFR mutations, such as surrogate markers, are necessary. CEA is the product of the CEACAM5 gene, which is expressed only in epithelial cells. CEA is found more abundantly on the apical surface of the gastrointestinal epithelium but is also found in other mucosal epithelia cells, such as in the lung [26]. Although CEA was often falsely elevated in smokers and in patients with restrictive or obstructive pulmonary diseases [27–29], abnormally elevated CEA levels were reported in 30-70% of patients with NSCLC. Abnormally elevated CEA levels are most frequently observed in patients with adenocarcinoma and advanced stage carcinoma [30]. In addition, high serum CEA levels are associated with a poor prognosis in patients with NSCLC, regardless of treatment [30, 31]. According to Japanese scholars, patients with elevated serum CEA levels responded better to gefitinib. Furthermore, recurrent lung adenocarcinoma patients with high serological CEA levels have a higher EGFR mutation rate after surgery and higher serological CEA levels. These findings are attributed to a possible anti-apoptotic signal in the mutant EGFR pathway that could elevate the expression level of the CEA protein [32]. However, the specimens used for genetic testing were surgical specimens obtained prior to disease recurrence and may not represent all the biological characteristics of a recurrent tumor [33]. In our study, the serum CEA level in the EGFR gene mutation group was significantly higher than in the non-mutated group. Both the univariate and multivariate analyses indicated that the serum CEA levels correlated with EGFR mutations (higher serum CEA levels were associated with higher EGFR gene mutation rates). Our data are similar to the findings of Okamato et al. [34]. Shoji et al. [35] reported that the rate of EGFR gene mutation significantly increased as the serum CEA levels increased (for serum CEA levels <5, ≥5 (but <20), and ≥20, the rates of EGFR gene mutation were 35, 55 and 87.5%, respectively; *p* = 0.040).

Several reports have described the relationship between serological markers and the curative effect of EGFR-TKIs. However, these reports did not perform EGFR mutation testing or dynamic monitoring of CEA levels to predict EGFR-TKI resistance. Therefore, these reports cannot determine the most effective treatment for early intervention. Despite the high responsiveness of tumors bearing activating EGFR mutations, almost all patients become resistant to TKIs. Multiple molecular mechanisms may underlie this resistance, including secondary EGFR mutations, bypassed signaling activation, and phenotypic transformation. Because multiple molecular mechanisms may lead to EGFR-TKI resistance, it is important to non-invasively detect tumors refractory to EGFR-TKI treatment and identify the mechanisms underlying this resistance. Thus, the therapy could be effectively tailored to each patient. Based on previous reports, the function of CEA has not been elucidated. However, as a cell surface adhesion protein, CEA may play a role in cell-cell adhesion [36]. Overexpression of CEA is thought to play a role in tumorigenesis [37]. Furthermore, CEA has a dominant effect in blocking differentiation, and CEA cooperates with Myc and Bcl-2 during cellular transformation [38]. Furthermore, CEA can inhibit cell death induced by a loss of anchorage to the extracellular matrix (anoikis) [39]. If CEA is upregulated following activation of the EGFR pathway, its serum levels may trigger an EGFR mutation. Although these findings suggest that CEA may have anti-apoptotic effects in cancer cells, a direct relationship between high CEA levels and patient responses to EGFR-TKIs has not yet been established and requires additional research.

We found that a persistently high level of CEA after treatment with a reversible EGFR-TKI can successfully identify patients with NSCLC cells that are resistant (perhaps because of the occurrence in the EGFR kinase domain of a T790 M secondary mutation that prevents EGFR-TKI binding and subsequent growth arrest). Furthermore, when the CEA level was 16.2 times greater than normal, the elevation was associated with distant metastasis (Table 2). According to Sequist et al. [39], molecular analyses of repeated lung biopsies from these patients are needed to identify different mechanisms of acquired resistance. A potential clinical application of our observations could be the development of a test for patient responsiveness to EGFR-TKI treatment using non-invasive serum tumor markers. The information provided by this test may facilitate the selection of patients as candidates for therapy with reversible or irreversible EGFR-TKIs and the development of therapeutic strategies for overcoming resistance in patients with refractory NSCLC. Tumors with high CEA expression may possess an increased capacity to develop distant metastases (perhaps due to vascular-tumoral cell-cell adhesion processes). CEA serum levels may identify patients with a high risk of metastasis development prior to CT scans. Other cell adhesion molecular markers associated with lymph node metastasis, such as the chemokine receptors CCR7, CXCR3 and CCL21, could be related to distant metastasis development. Thus, studies of their association with distant metastasis development are justified.

In our study, the OS-associated factors were age, clinical stage, and serum CEA levels. In many neoplasms, a high serum CEA level predicts residual disease or tumor relapse in patients without normal-range serum levels after surgery [40]. In fact, Iwasaki et al. proposed a formula to evaluate mortality risk based on CEA serum levels, histological type, and the presence of positive mediastinal lymph nodes [41] High CEA serum levels may reflect micrometastatic disease, although we detected no differences in the CEA serum levels between patients of different clinical stages. This observation suggests that the prognostic role of high CEA serum levels may be completely accounted for by tumor change. CEA represents an important tumor marker associated with several physiopathological CEA expression is induced by hypoxia inducible factor α (HIF-α), suggesting that CEA plays an important role as a micro-environmental factor during tumorigenesis and confers a worse prognosis.

To our knowledge, the clinical assessment of lung cancer treatment uses the RECIST criteria as the gold standard for response evaluations. However, early diagnostic CT scans for response evaluations in patients receiving EGFR-TKI therapies have severe limitations. EGFR-TKI therapy is expected to induce a response via cytostasis, rather than an objective morphologic response. The RECIST criteria are further confounded by structural abnormalities, both before and after treatment, which may not actually be tumors.

The limitations of this study should be acknowledged. There is no consensus regarding the optimal timing for performing either CT scans or serum CEA measurements during or after prolonged treatments. According to RECIST version 1.1, the best radiologic response evaluation can be obtained at least 4 weeks after the initiation of therapy. In our study, we performed CT scans every 2 weeks after the initiation of therapy. Therefore, the relatively small number of patients exhibiting a radiologic response could be explained by the timing of the CT scans. In addition, normal serum CEA levels in this study ranged from 0.0 to 3.4 ng/ml, which are lower than the previously reported value of 5.0 ng/ml [27, 42, 43].

## Conclusion

Patients with elevated serum CEA levels responded more positively to EGFR-TKIs, and lung adenocarcinoma patients with high serological CEA levels exhibited a higher rate of EGFR mutations.

In addition, we found that serum CEA levels several times higher than normal upon diagnosis was an independent prognostic factor for metastasis development – particularly to the brain, liver, adrenal gland and other distant viscera – over a short time frame in patients undergoing treatment with EGFR-TKIs. Thus, patients with EGFR-TKI-resistance should undergo combined chemotherapy and radiotherapy. The feasibility of new diagnostic techniques will improve the understanding of EGFR-TKI resistance. Therefore, we believe that CEA represents a potential molecular target.

## Abbreviations

CEA: Serum carcinoembryonic antigen
cftDNA: cell-free tumor DNA
CT: Computed tomography
EGFR: Epidermal growth factor receptor
EGFR-TKI: Epidermal growth factor receptor tyrosine kinase inhibitor
NSCLC: Non-small cell lung cancer
RT-PCR: Real-time quantitative PCR
